# Structural parameter variation working on the performance of anti-uplift multibell underreamed anchors

**DOI:** 10.1038/s41598-022-19353-8

**Published:** 2022-09-02

**Authors:** Chen Chen, Yuanyou Xia, Manqing Lin, Qing Ni

**Affiliations:** 1 School of Civil Engineering and Architecture, Wuhan Institute of Technology, Wuhan, 430073 China; 2 School of Civil Engineering and Architecture, Wuhan University of Technology, Wuhan, 430070 China; 3 School of Resources and Safety Engineering, Wuhan Institute of Technology, Wuhan, 430073 China; 4 School of Engineering, University of Warwick, Coventry, CV4 7ES UK

**Keywords:** Civil engineering, Engineering

## Abstract

The structural parameters of multibell underreamed anchors play a crucial role in anchoring performance. The parameters of multibell underreamed anchor investigations are helpful when exploring optimized anchor structures. Based on the results of small-scale physical modelling tests, two types of multibell underreamed anchors were adopted under vertical uplifting loads. Numerical investigations were employed to study the effect of bell spacing, underream structure and bell dimension on the ultimate uplift bearing capacity. After an analysis of the anchorage mechanism, the anchoring efficiency was evaluated by the anchoring force provided by the unit concrete usage of the anchor, and the structural parameter *λ* equal to the surface area ratios of the expanded bell cone to the straight shaft between bells was defined. Then, the anchoring efficiency optimized structural parameters were presented. An analysis of model tests and simulation results showed that compared to concave bell surfaces, the convex shape could enhance the ultimate bearing capacity of a multibell underreamed anchor. There is an optimal value for the spacing of neighbouring bells, and there are three models of mechanisms for multibell anchors pulling out. When *λ* ∈ [1, 1.8], the multibell anchors can perform most efficiently to achieve their structural advantages.

## Introduction

Multibell underreamed anchors are generally employed in pipe grouting technology to form a series of bells or enlarged underreams in the anchorage section. The instruments, construction steps and anchor size are clearly stipulated in the standards^1,2^. Multiple enlarged underreamed anchors are used in fine-grained soil, which can effectively improve the anchoring force. Because of their unique advantages, these anchors been gradually introduced into all kinds of engineering projects and have been written into the engineering codes of many countries^1,2^.

Underreamed ground anchors, such as multiple underreamed anchors, are usually applied in soft fine-grained soil or clay to improve uplift capacity^3^. Shear resistance inside the soil, transferring through the soil–structure surface, makes up the anchorage bearing capacity, and this capacity is an important index used to evaluate the anchorage effect^4^. For underreamed anchors, the end bearing resistance on the “shoulder” of the anchor can enhance the uplift load more efficiently. The anchorage mechanism of an underreamed anchor with one enlargement has already been thoroughly studied^5–8^.

The working mechanisms of underreamed piles and anchors are similar. Anchor burial depth was investigated by model tests^7^, and the results showed that the bearing capacity of an anchor ‘shoulder’ accounts for more than 60% of the total uplift load, regardless of a shallow or deep burial mode. In particular, ruptures seemed more likely to occur around the enlargement area in the shallow burial scenario; however, the influence of various underream dimensions was not discussed. The failure modes in shallow and deep burial were observed to show great differences in Hsu’s experiment^8^. In the case of uplifting a shallow anchor to its ultimate load, the yield soils extended to the ground surface, which did not occur for a deep anchor. Later, an introduced SHASOVOD model, together with FLAC 3D software, was adopted to carry out investigations on arrayed anchor groups, and anchorages were found to be more efficient under deeper burial conditions. The British code^2^ simplified the multibell enlargements into one single expansion segment when calculating the ultimate uplift capacity of an anchor buried deeply. However, the author's previous research found that this method was conservative and neglected the influence of the end bearing force of each bell. By changing the relationships among the shaft diameter, bell diameter and bell spacing, there is an optimal combination, which can effectively liberate the end bearing capacity of each bell and achieve the optimal anchorage efficiency of a multi-underreamed anchor^9^.

Compared with a single enlarged anchor, a multibell underreamed anchor can significantly improve the uplift capacity^10^, and the influence of burial depth cannot be ignored^11^. Scholars have studied the position of the enlarged body on the anchor^12^, the enlargement shape^13^ and the underreamed size^14,15^, but these factors have not been comprehensively considered. Although few studies have focused on multi-underreamed anchors, other basic investigations have been conducted on plate anchors or screw anchors, which are similar in their anchorage mechanisms. The ultimate uplift capacity of an anchor can be greatly improved by increasing the number of anchor discs^16^, but this approach has limits. If there are more than three anchor discs, the improvement is not obvious. The optimal spacing of anchor discs is 24.5–35.5 mm^17^. By comparing screw anchors with underreamed anchors in the same diameter, it was found that the surface friction of the screw anchor was greater^18^. When the soil cohesion value was larger than 60 kPa, the ultimate uplift capacity of the screw anchor was greater than that of the underreamed anchor; when it was below 60 kPa, the scenario was the opposite^19^. However, screw anchors are disadvantaged by shallow burial depths and additional requirements for installation rigs, which makes it difficult to satisfy the needs of some projects^20^.

A transparent soil test can be used to observe the interaction between the underream anchorage and the soil when the anchor is pulled out upwards^21^. Multibell underream anchors are one type of multi-enlargement anchor; although they are rarely used in China, Western countries have several decades of experience in their application^2^. The results from the author’s previous studies of transparent soil model tests^22^ showed that multibell anchors could greatly enhance uplift bearing capacities by approximately 66–91% compared to traditional anchors. This approach could also promote anchorage efficiency due to its end-bearing capacity, thereby reducing concrete usage.

The intention of this paper is to study a model experiment of multibell underreamed anchors pulling out in fine-grained soil or clay based on transparent soil and the particle image velocimetry (PIV) technique. Then, numerical simulation analysis was employed to investigate the anchorage mechanism and the effect of different dimensions and distances of neighbouring bells on underreamed anchors. In the physical modelling tests, two types of anchors, D1^22^ and D2, were chosen, with different boreholes and enlargement diameters, for pull-out tests. Then, numerical simulations focused on bell patterns and dimensions of the anchor were employed to determine the effect on the ultimate bearing capacity of anchors. Finally, the optimal structure layout of multibell underreamed anchors was discussed. The results can be used as a reference for the design of similar engineering.

## Model test

**Figure 1:**
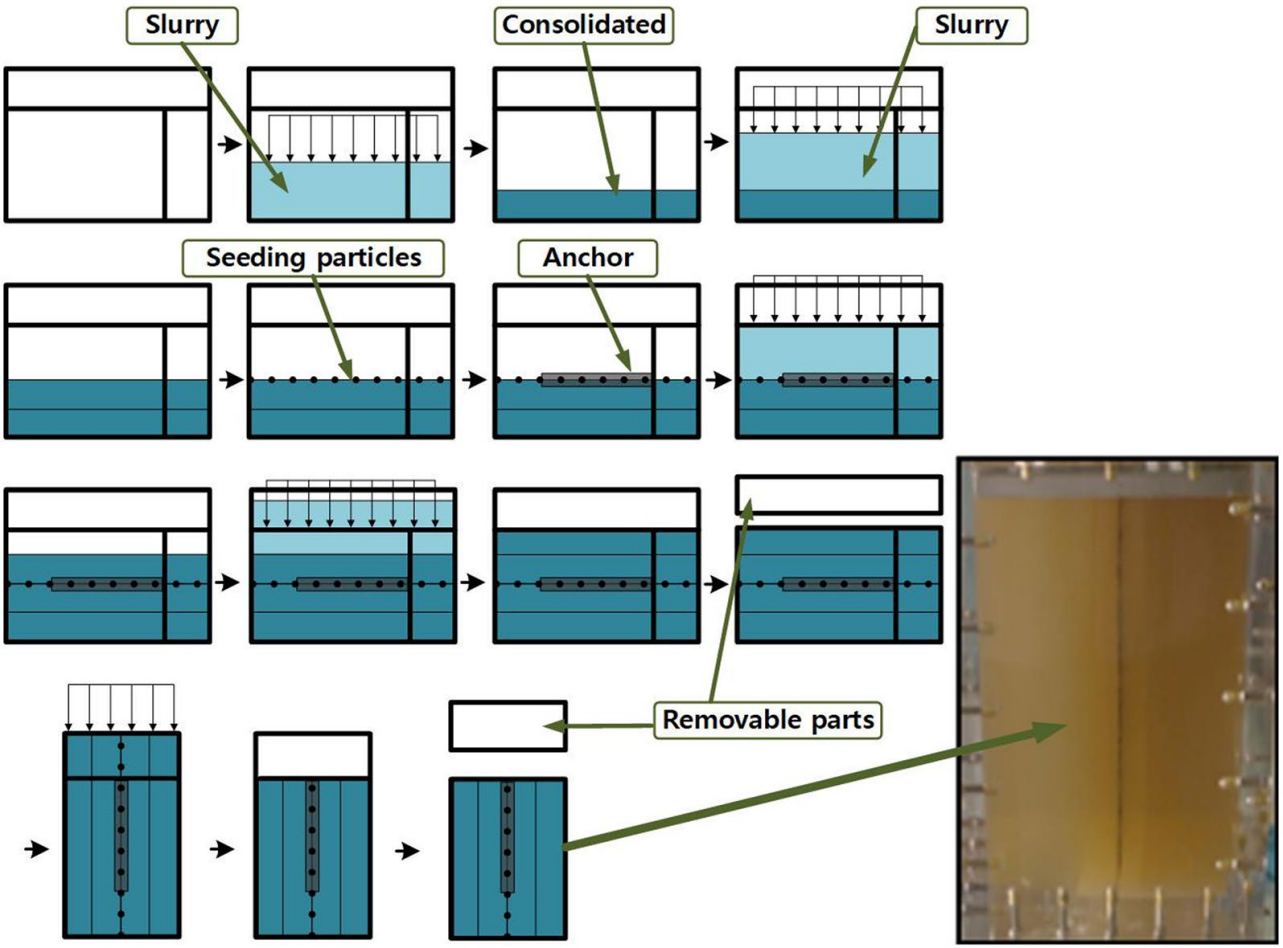
Transparent soil consolidation.

To simulate clay^23^ or fine-grained soils^24^, transparent soils use a mixture of mineral oil and amorphous silica to conduct stepwise consolidation^25^. The preparation scheme of transparent soil samples in this test is consistent with Dr. Hover’s^25^, and various related parameters of transparent soil have been studied extensively by many scholars^25–27^. The physical parameters of the transparent soil are shown in Table 1. Transparent soil samples first consolidate horizontally. The first step is to place the Perspex box horizontally, pour in a layer of the transparent soil slurry for the first consolidation, and then follow with a second layer. After two layers of transparent soil consolidations are accomplished, the sample surface reaches the middle of the box. At this point, seeding particles and anchor embedment are carried out. After that, the third and fourth layers of consolidation follow. After horizontal consolidation, the excess extension of the box is removed and sealed before vertical consolidation. Step-by-step vertical consolidation loading eventually reaches 40 kPa, and then the extended part of the box is removed for testing, as shown in Fig. 1.

**Table 1:** Properties of transparent soil.

Refractive index (at 24℃)	Density (kg/m^3^)	Young’s modulus, undrained/MPa	Poisson’s ratio	OCR	$c$/KPa	*φ*/(°)
1.447	1572	3–19	0.45–0.5	1	5.93	36

**Figure 2:**
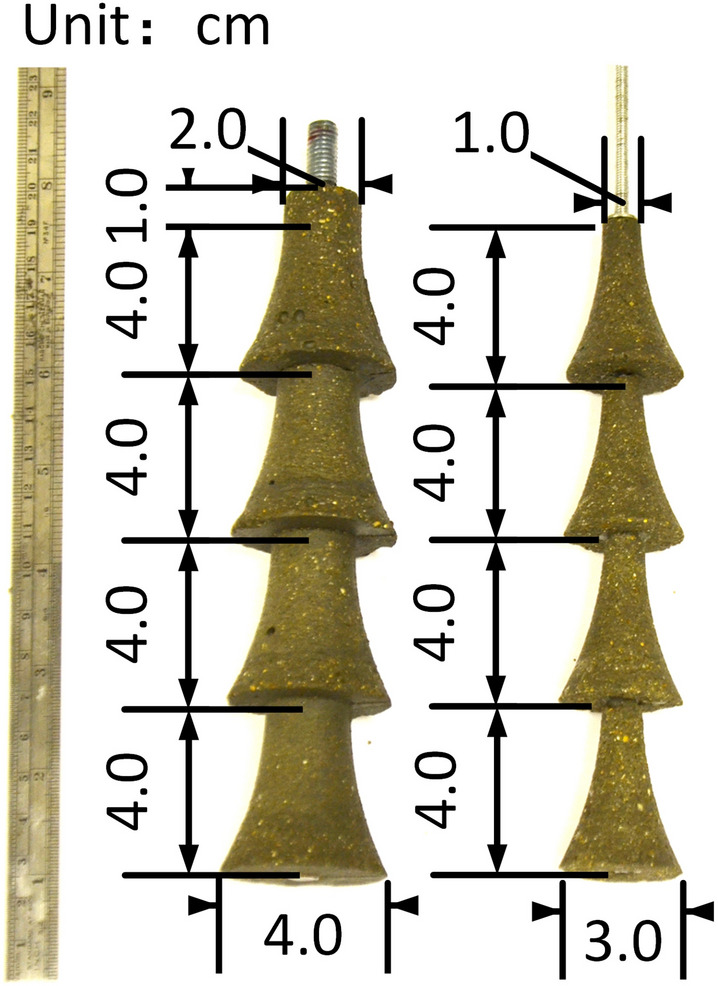
Multibell anchors for tests.

**Figure 3:**
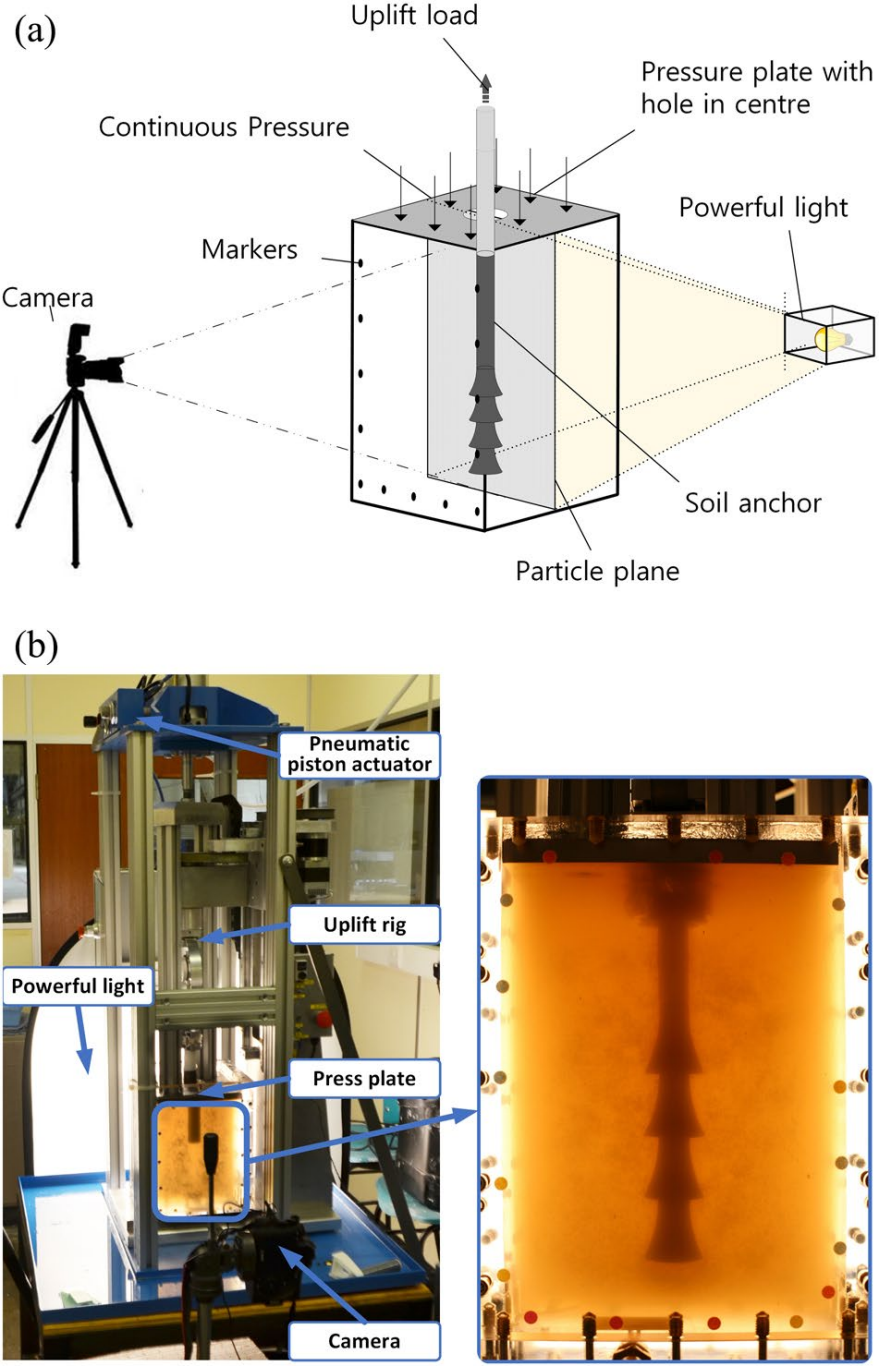
Photo of physical model tests. (**a**) Experiment setup. (**b**) Real photo of the model test.

During the test, the soil top surface is subjected to a constant uniformly distributed pressure of 40 kPa to simulate deep burial. The size of the Perspex box is 20 × 20 × 30 cm. Test anchors are fabricated with concrete and steel rods at a ratio of 1:10. Details of the model anchors and the dimensions for each part are shown in Fig. 2. D1 is shown on the left and D2 on the right. Two anchors have borehole diameters of 2 cm and 1 cm and bell diameters of 4 cm and 3 cm for D1 and D2, respectively. The transparent soil tests were carried out in the transparent soil laboratory of the University of Warwick, UK, and setup details can be found in Fig. 3. Figure 3a and Fig. 3b show the model test setup and real photo of the model test, respectively. The anchor uplifting speed was set at 13.8 mm/min^25^.

## Model test results and analysis

**Figure 4:**
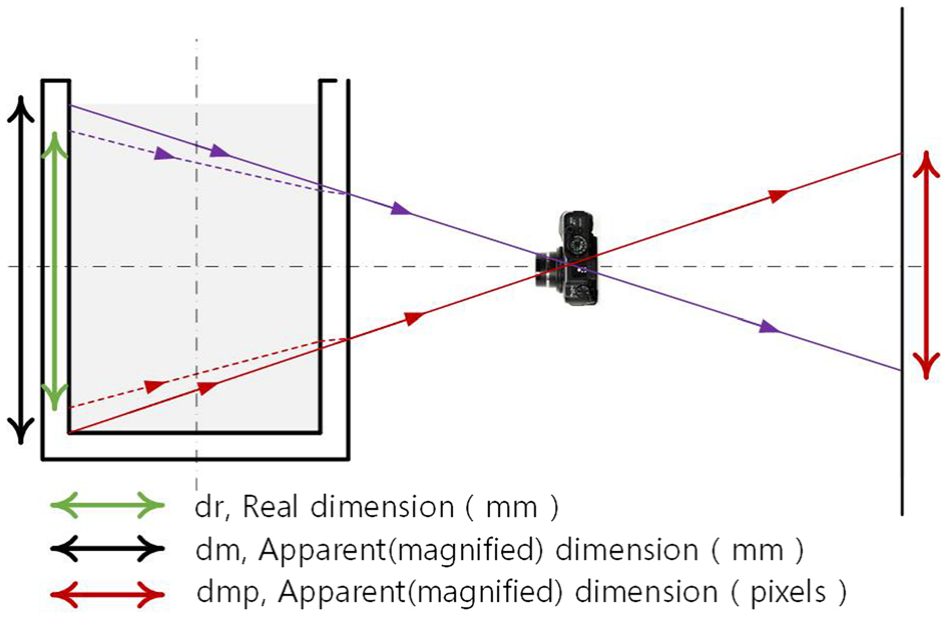
Effects of refraction.

The experimental image acquisition equipment was a DSLR camera, and the shooting rate was kept at 4.5 frames/s. After the transparent soil area of the subsequent images was divided into interrogated patches with a definition of 50 × 50 pixels, the vertical displacements of the soil were processed and analysed using the PIV technique. Because light refraction through the various transparent materials can cause distortion, the plane behind the materials has been magnified by light bending as it meets each boundary between materials, the principle of which is shown in Fig. 4. As shown in Fig. 4, the distortion caused by refraction should have a linear correlation with the shooting distance; simply speaking, this is magnification. In this magnification, the coordinates of each point, each displacement, and each dimension are scaled up by the same factor, meaning that the spacing used to reconstruct the coordinates of all marker points is the same spacing as that between all markers in the image. Based on this linear relationship, the sole data correction required for magnification is that of a linear scale factor (LSF) to convert pixels to mm. The method measures the ratio between the size of an object (mm) and the pixel size of an object in an image. Then, the average can be taken through multiple trials. The amplification of the LSF due to light refraction was determined to be 1.05 through a series of tests.

**Figure 5:**
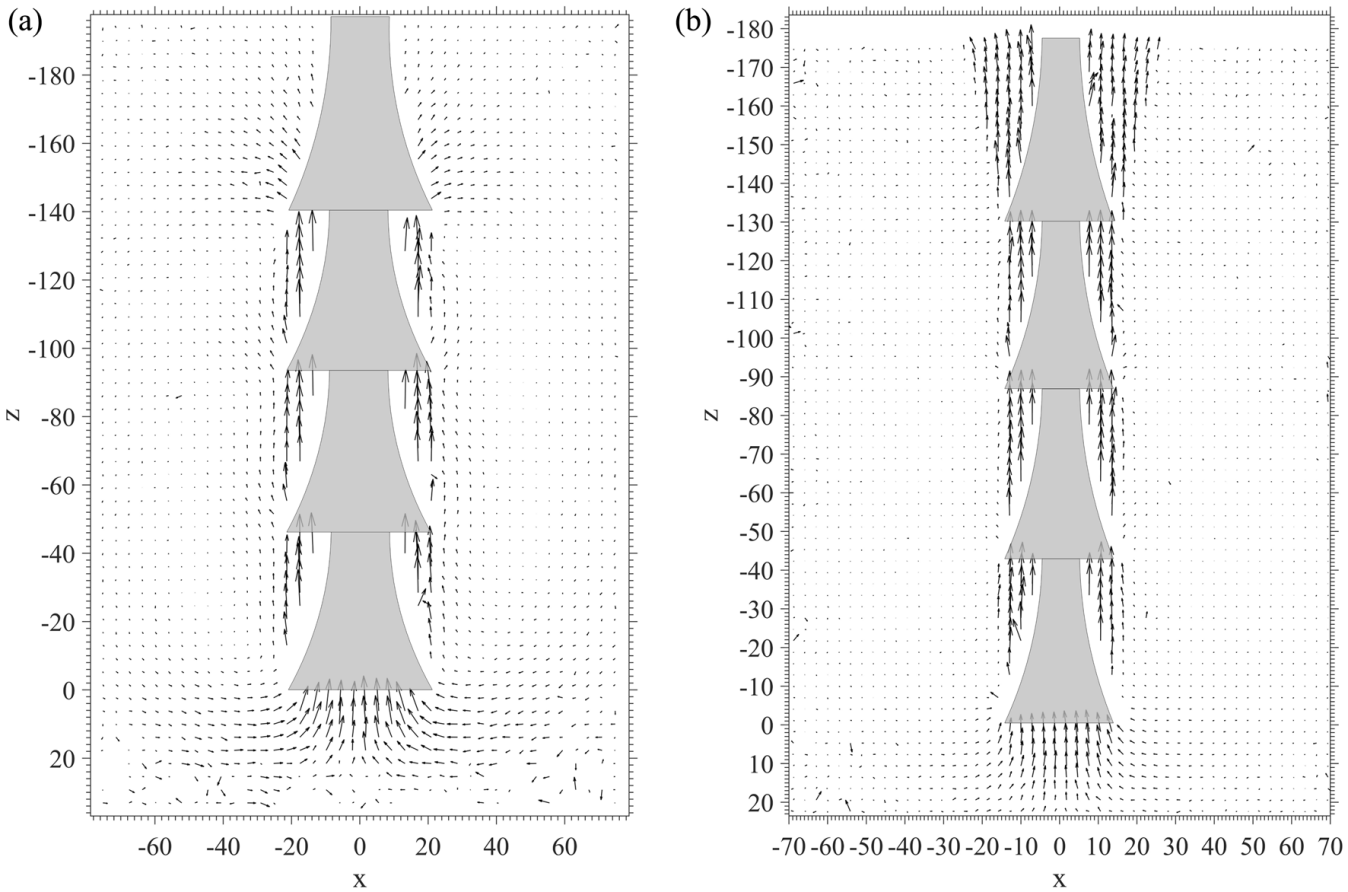
Displacement vectors around anchorage under pulling process. (**a**) D1. (**b**) D2.

**Figure 6:**
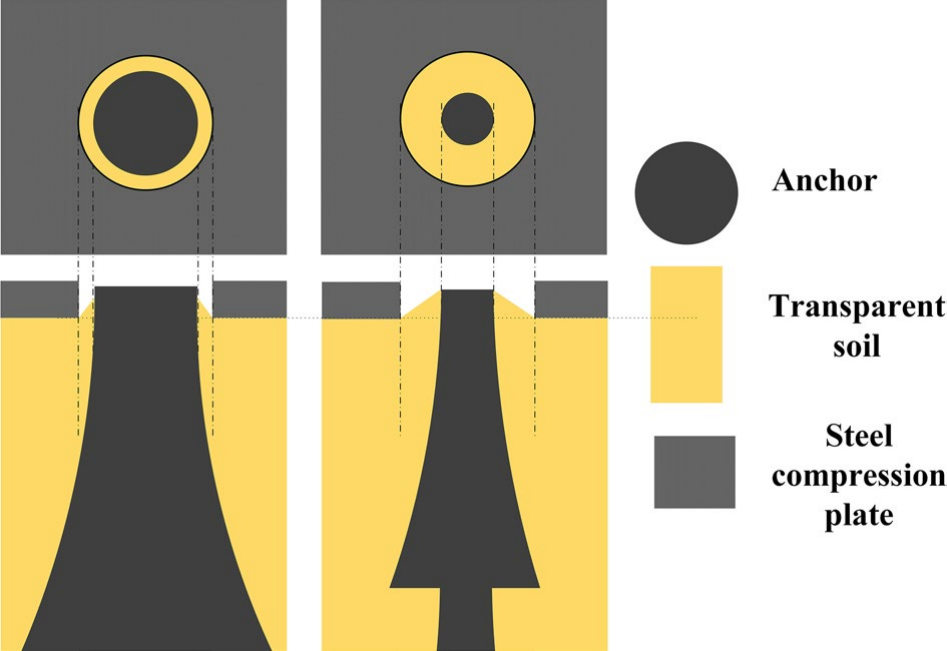
Explanations for different vertical soil movement near the hole of pressure plate.

The time period from when the anchor is being uplifted to when it reaches the ultimate bearing capacity is defined as *t*. The displacement vectors of the surrounding soil were plotted using arrows. The displacement vector distributions of anchor types D1 and D2 are shown in Fig. 5a,b. In Fig. 5, it can be seen that during *t*, both D1 and D2 showed similar displacement patterns: cylindrical potential failure surfaces were formed close to the underreams. However, the soil displacement around the shaft on the top underream of D2 was obviously greater than that around D1. There were two explanations for this: on the one hand, the shaft above the underream of D1 was 1 cm longer than D2; on the other hand, the smaller shaft diameter of D2 could allow more soil to escape than D1 when uplifted through the same hole on the pressure plate, as shown in Fig. 6. Conversely, the soil displacements at the anchor bottom of D1 were larger than those of D2. This was because when the soil anchor was being pulled out, the larger anchor needed more soil to fill the cavity created at the bottom.

**Figure 7:**
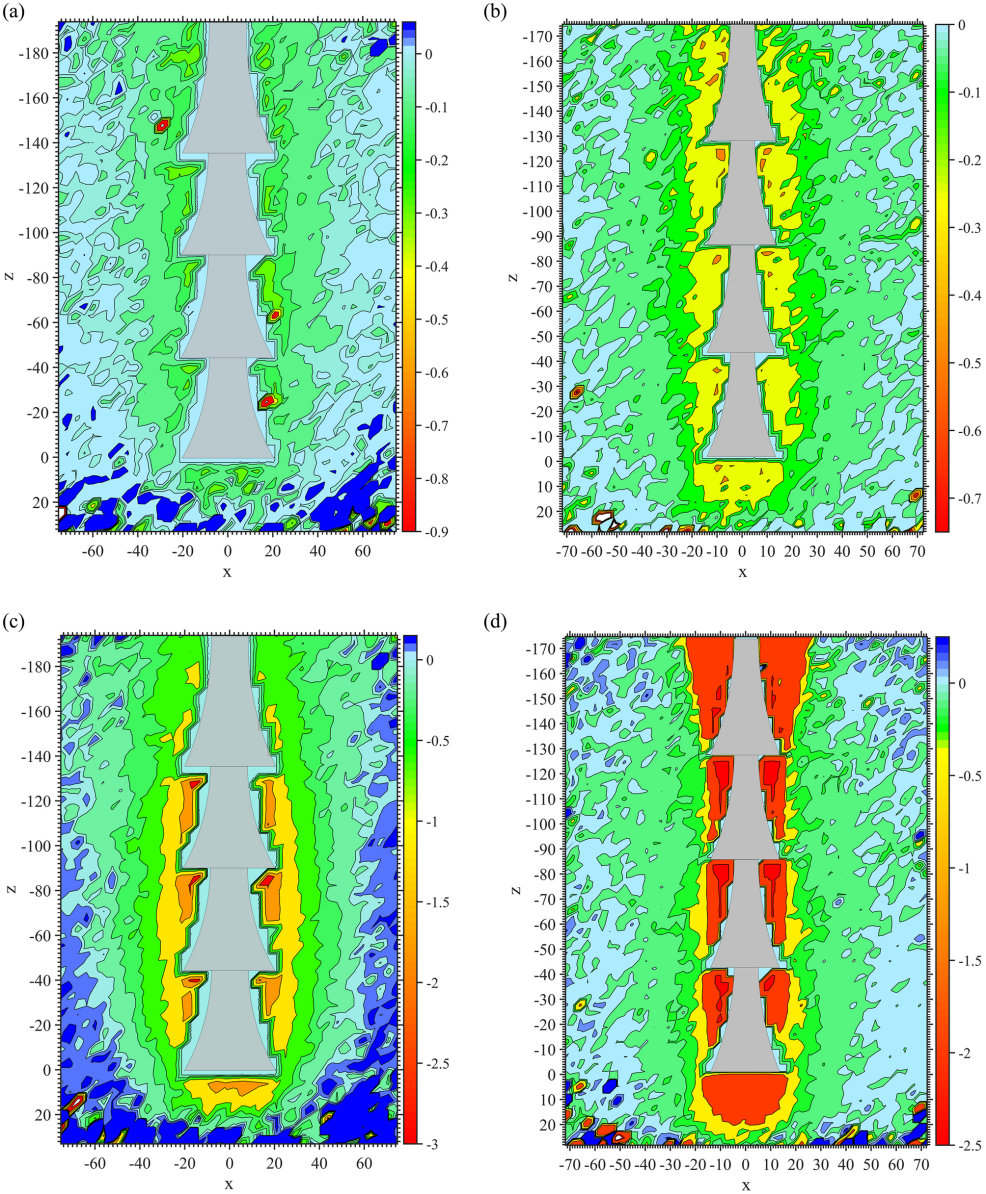

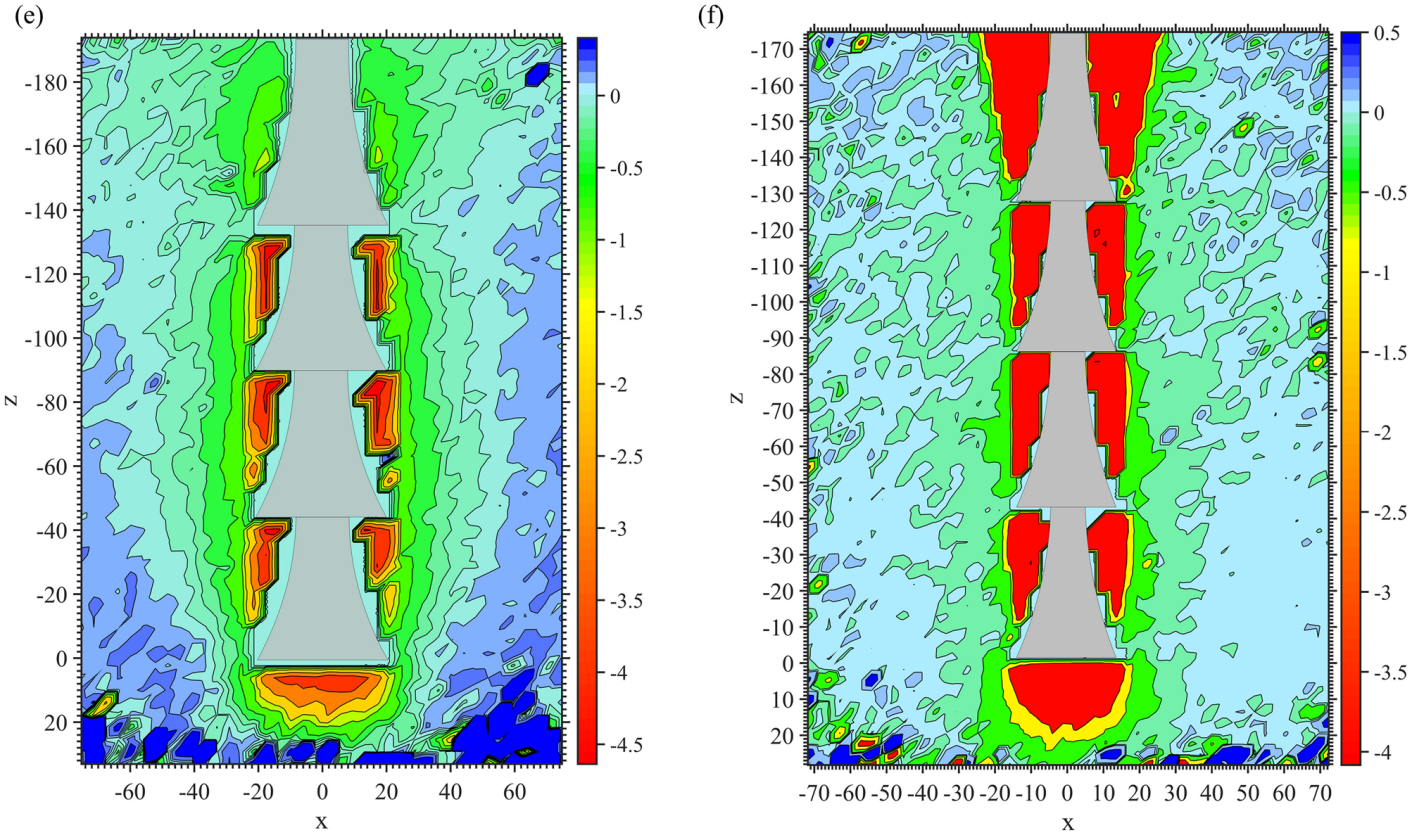
Soil displacement contours (mm). (**a**) D1 0.2*t*. (**b**) D2 0.2*t*. (**c**) D1 0.6*t*. (**d**) D2 0.6*t*. (**e**) D1 1.0*t*. (**f**) D2 1.0*t*.

The time to failure *t* for each test was determined, and vertical soil displacement contours were obtained at various stages of the test, as shown in Fig. 7. The spindle pattern of the soil displacement contours was observed from 0.2*t* in both Fig. 7a,b. Inevitably, several noise spots were generated, caused by relatively smaller soil displacements at 0.2*t*. During the time period between 0.6*t* and 1.0*t*, the soil displacement contours close to the shaft near the top underream of D2 were more extensive than those of D1, which could also be explained by the same reason previously mentioned (Fig. 6). The maximum soil vertical displacements crowded at the concave and bottom of the underreams. This was caused by the vacuum space created by uplifting anchors so that the soil moved to fill the space consequently, induced by the pressure from the steel plate and dead weight of the soil. At 0.6*t* and 1.0*t*, the soil vertical contours of D1 still maintained the spindle shape.

**Figure 8:**
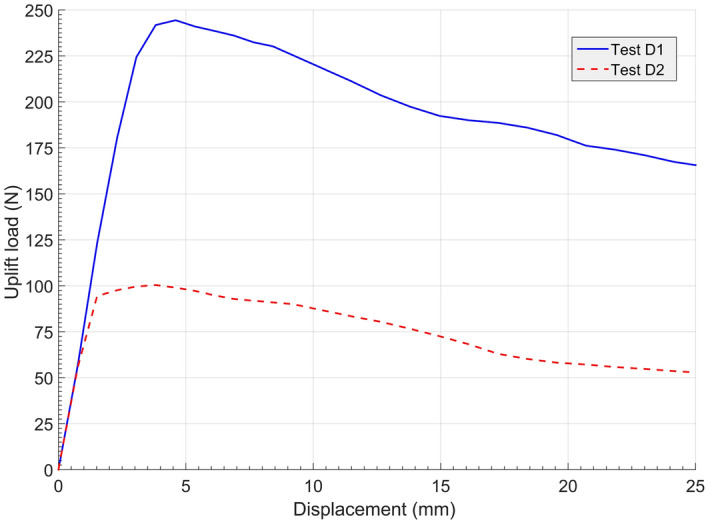
Uplift load–displacement curves of soil anchors.

The variation of the two curves tendency can be seen clearly in Fig. 8, and the uplift bearing capacity of D1 is nearly twice as much as that of D2. The two load‒displacement curves keep increasing almost linearly and nearly intersect until reaching their own peaks at 1.0*t*. While the uplift load of type D2 reached its peak, the uplift load for type D1 continued to increase, leading to different maximum load and ultimate capacity values. Figure 7 shows that the type D1 anchor was stronger and perhaps more durable than type D2 under the same pulling out speed, as shown in Table 2. After reaching their own summits, both curves decrease rapidly and then slow down into residual strength.

**Table 2:** Maximum uplift loads and failure time of soil anchors.

Anchor type	D1	D2
Ultimate load displacement (mm)	4.4	3.6
Ultimate bearing capacity (N)	244.8	135.7
Standard calculation (N)	234.6	156.4
Error (%)	4.2	− 15.3

A comparison of ultimate uplift bearing capacity from model tests and standards are presented in Table 2. Table 2 implies that the ultimate bearing capacity of anchor type D1 was 80.4% greater than type D2, but it required 0.8 mm more displacement to reach that capacity.

In Table 2, it can be seen that the standard calculation for type D1 was closer to the test results. This could still be explained by Fig. 6. The equation in the standards suggests that the potential fracture surface close to the underreams was considered a cylindrical surface, which was coincidently the same as the contour plots obtained from the model test observation in Fig. 5. In this case, the calculations were very close to the test results. However, the reason why the anchorage mechanism behaved like this was due to the short spacing of the bells, which also meant each bell did not work independently. Early research by the author's team on a multiball anchor^9^ showed that an increase in the enlargement distance could improve the ultimate bearing capacity. Finding the optimal structure of multibell anchors was the main goal of the following investigation.

## Numerical simulation analysis of the ultimate uplift bearing capacity influenced by variations in the anchorage structural parameters

**Figure 9:**
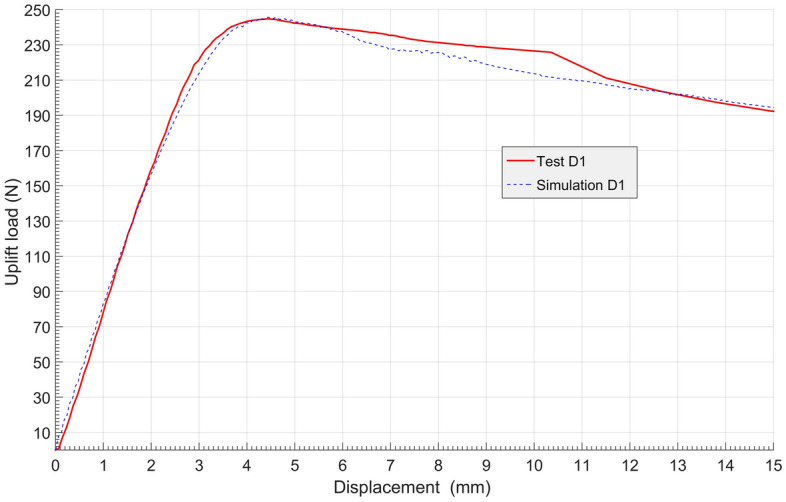
Comparison of D1 uplift load–displacement curves between model test and simulation.

Flac 3D finite difference numerical simulation software^24,28^ was used for numerical simulation, and the simulation parameters were set strictly in accordance with the model test conditions. First, the accuracy of the numerical simulation was verified with a model test^24^. The Mohr‒Coulomb model was used for the constitutive model, and the elastic model was used for the anchorage section and the press plate. The interface elements were established between the soil and the anchorage section and between the soil and the pressure plate. The load‒displacement curves of both the model test and numerical simulation are shown in Fig. 9. Figure 9 shows that the consistency between the model test and numerical simulation was quite good. The results show that numerical simulation can peplicate the process of the model test well.

### The influence of different bell shapes on ultimate uplift bearing capacity (Group M1)

**Figure 10:**
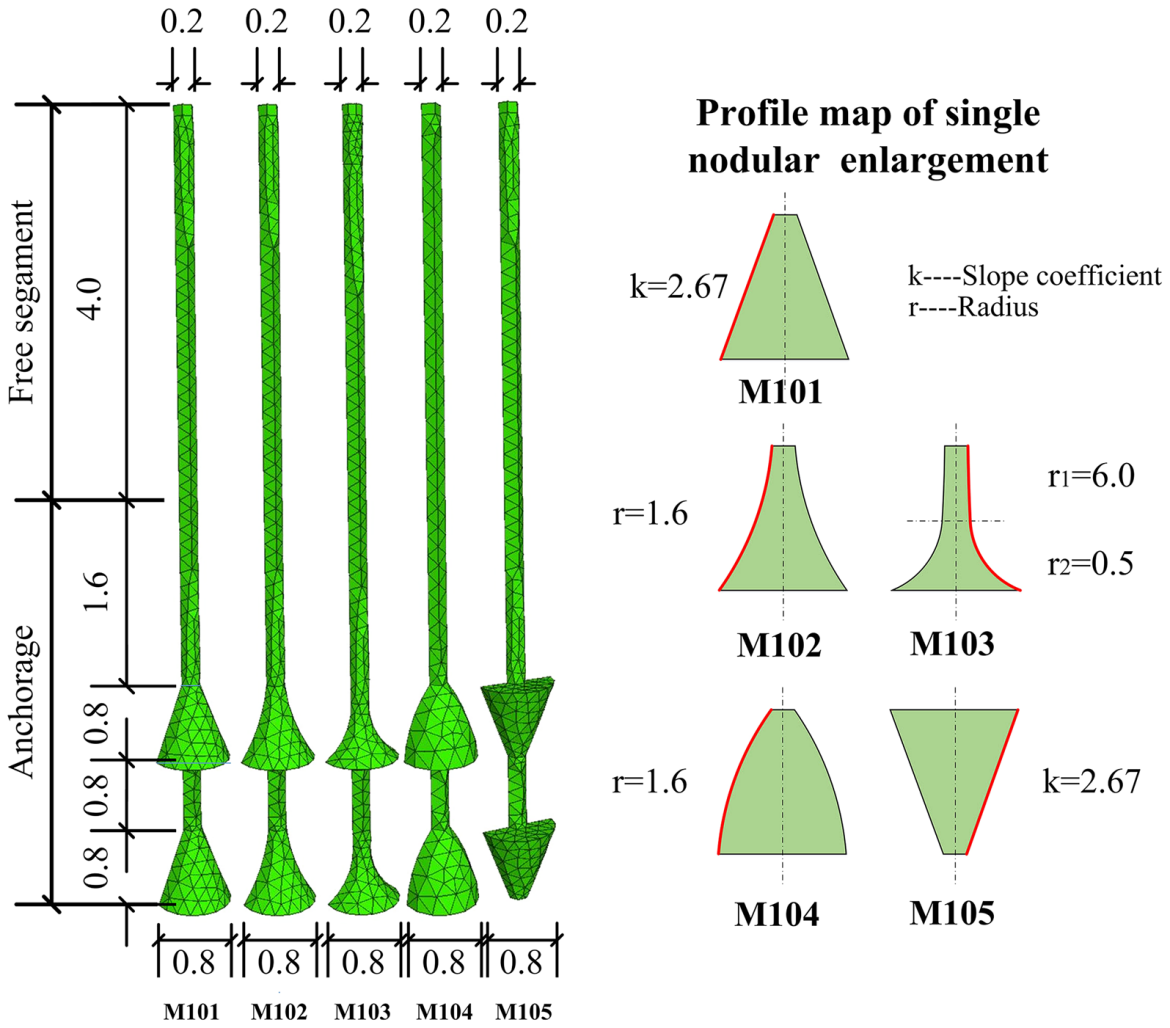
Diagram of anchors in group M1 (Unit: m).

Mickovski and Ennos^29^ carried out interesting experiments on the resistance of plant roots to uplift loading. The plant roots were cylindrical, conical, bulb, spherical, inverted cone, and inverted bulb shapes, with 6 types in total. The test results indicated that conical roots showed stronger resistance to uplift than cylindrical or spherical bulbs. However, all the root systems were shallowly buried and at small scales. In this section, 4 different concave–convex bell surfaces plus 1 inverted conical were used in the numerical simulations. For the diagram of group M1, please see Fig. 10, where *k* is the slope coefficient and *r* is the curve radius. It is necessary to note that the cross-section curve of M103 was two arcs with different radii linked together.

**Figure 11:**
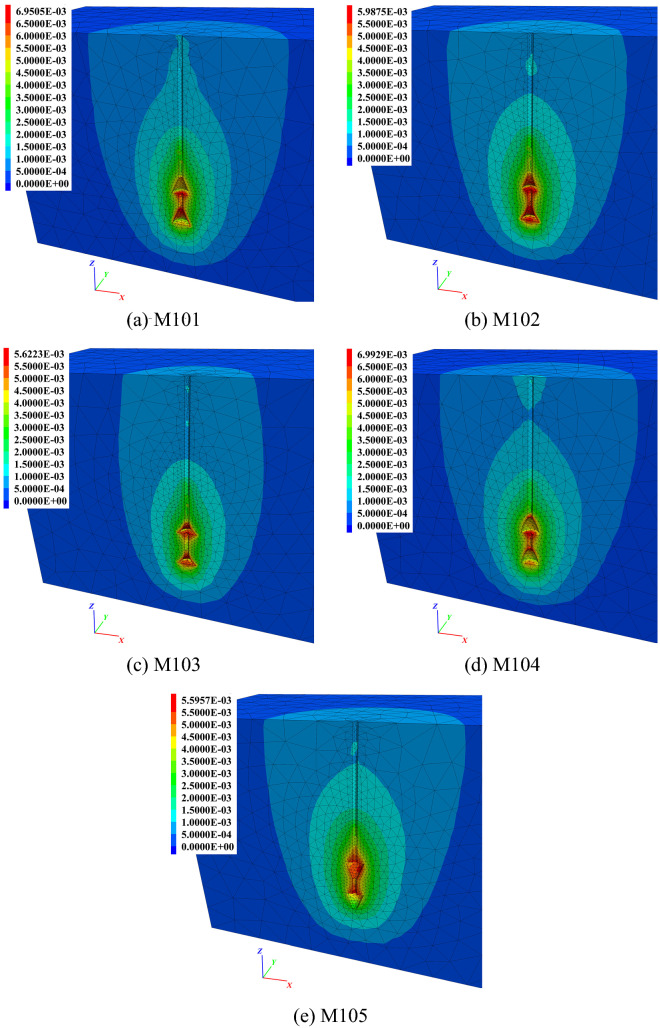
Soil vertical displacement contours of group M1 at 1.0*t*. (**a**) M101. (**b**) M102. (**c**) M103. (**d**) M104. (**e**) M105.

The soil vertical displacement contours of the 5 anchors are shown in Fig. 11. The red area in the figure represents the maximum soil vertical displacement. The shafts above the top bell of other anchors were not covered by red or yellow, indicating that before reaching 1.0*t*, those parts did not participate in resistance or failure occurred during the early stage of uplifting. However, due to the inverted conical shape of M105, the end bearing effect from the ‘shoulder’ affected the top shaft.

There were two anchors with parts of their underreams not covered by red or yellow colour. One was the middle shaft between the bells of M103 (Fig. 11c), and the other was the bottom inverted cone of M105 (Fig. 11e). The reason for M103 was that the increased curvature of its concave surface prolonged the shaft between neighbouring bells. This also weakened the ‘shoulder’ effect of the bells. For M105, the inverted conical surface could not form an effective anti-sliding mechanism, leading to a detached soil/grout interface at the beginning of anchor pulling out.

With the same bell height, increased curvature for the concave surface led to a smaller soil impact area close to the shaft between neighbouring bells. Taking anchors M101, M102 and M103 as examples, the boundaries of light yellow colour at this area were smaller one after another. It can be seen from M101 to M104 that the more concave the bell surface was, the more soil would stick to the bells when the anchors were being pulled out. For example, there were dark red regions on the bell concave space of M102 and M103. However, only parts of the bells were wrapped in red, as for M101 and M104. Overall, if the boundary of the light yellow area was taken as the potential fracture interface, then the largest area occurred in M104, especially located in the space between neighbouring bells. The light yellow areas of M101, M102 and M103 decreased successively.

**Figure 12:**
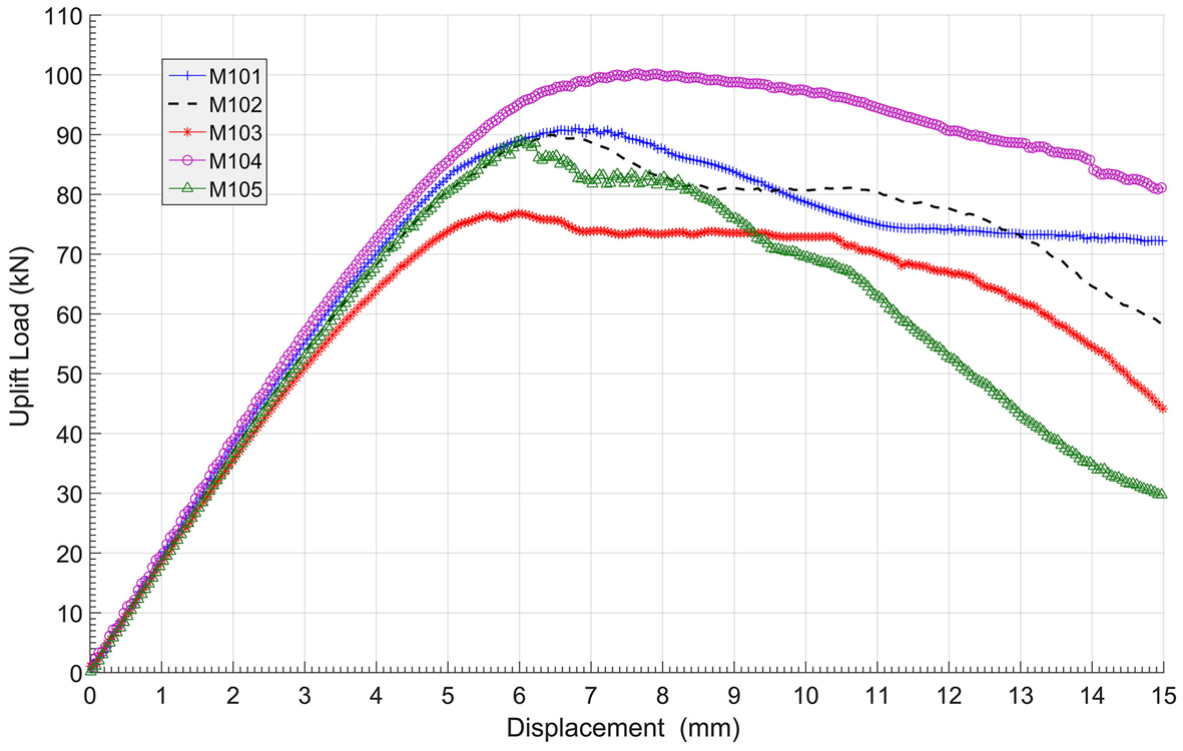
Load–displacement curves of group M1.

The load–displacement curves of the 5 anchors in group M1 are shown in Fig. 12. The figure shows that the ultimate bearing capacity of each anchor is strongly correlated to the size of its impact area, with an order of M104 > M101 > M102 > M105 > M103. All load–displacement curves behaved in similar patterns, increasing almost linearly first and slowing down in the development of resistance before reaching their summits, except for M105. After reaching its peak, the decrease in M105 was much more rapid than the others. This implies that the inverted conical structure was weak in maintaining the residual resistance of anchor uplift. The details of each ultimate uplift bearing capacity and each durable displacement are shown in Table 3. Neglecting M105 in Table 3, it can be concluded that the ultimate uplift bearing capacity and durable displacement can be increased by changing the surface of the bell from concave to convex. Furthermore, it can also be speculated that a better design of the conical surface of the bell could enhance the ultimate bearing capacity of the multibell anchor to approximately 33.2%.

**Table 3:** Ultimate uplift bearing capacities and vertical displacement of group M1.

Anchors	Ultimate uplift bearing capacities (kN)	Anchor vertical displacement (mm)
M101	89.0	7.4
M102	90.0	6.4
M103	76.8	6.0
M104	102.3	7.6
M105	88.9	6.0

The ultimate bearing capacity of each anchor, normalized by the concrete volume used, was calculated to represent the anchorage efficiency, as shown in Table 4. The anchorage efficiency of 5 anchors in group M1 could be aligned in the following order: M103 > M102 > M101 > M105 > M104. That is, in addition to M105, the increase in concrete usage might lower the anchorage efficiency. In other words, with the same borehole and underream diameter, reducing the amount of concrete used could boost the anchorage efficiency.

**Table 4:** Anchorage efficiency of group M1.

Anchor no.	M101	M102	M103	M104	M105
Concrete volume (m^3^)	0.211	0.168	0.119	0.270	0.211
Anchorage efficiency (kN/m^3^)	422.0	535.7	645.4	378.9	421.3

From the results above, it can be seen that when the anchor reached the ultimate bearing capacity, the upper shaft entered the relative sliding stage, namely, the residual shear strength stage, so its contribution to the bearing capacity was very small. The inverted conical setting of underreams was not helpful in promoting ultimate bearing capacity and was also inefficient in sustaining the residual resistance of the anchor after failure.

### The influence of bell spacing variation on ultimate uplift bearing capacity (Group M2)

**Figure 13:**
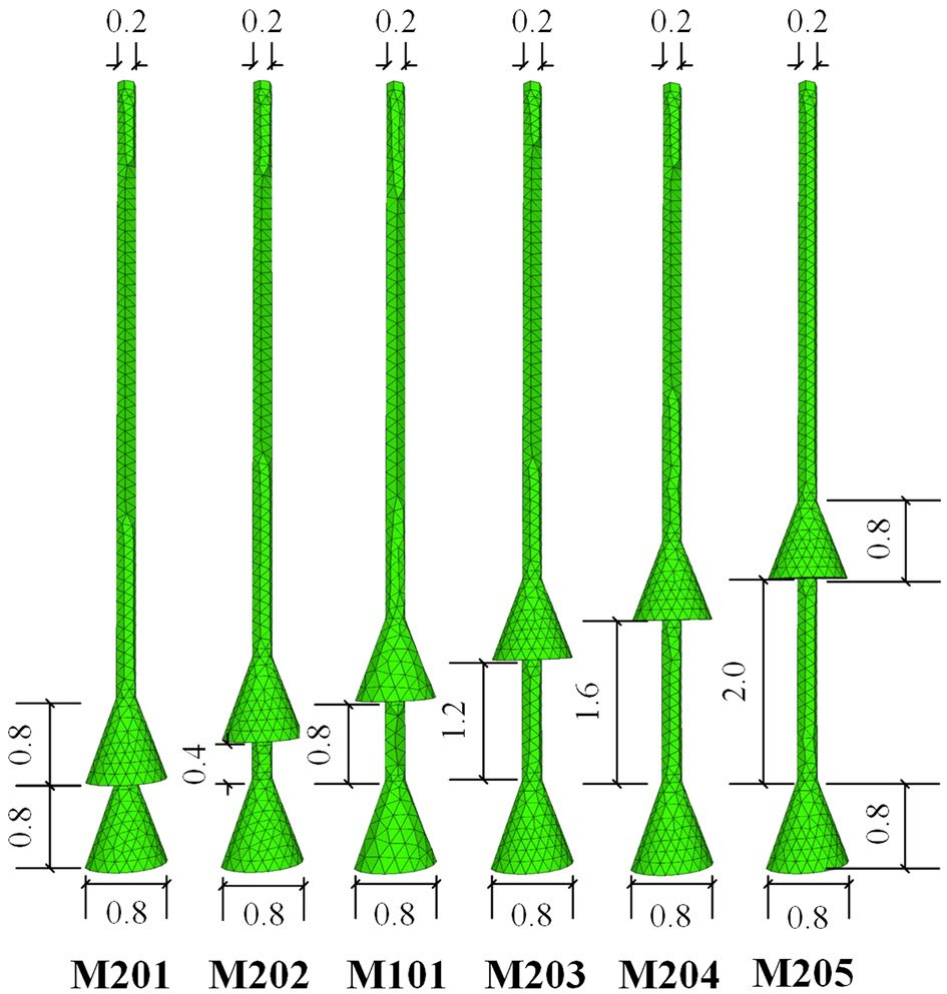
Diagram of group M2 anchors (Unit: m).

**Figure 14:**
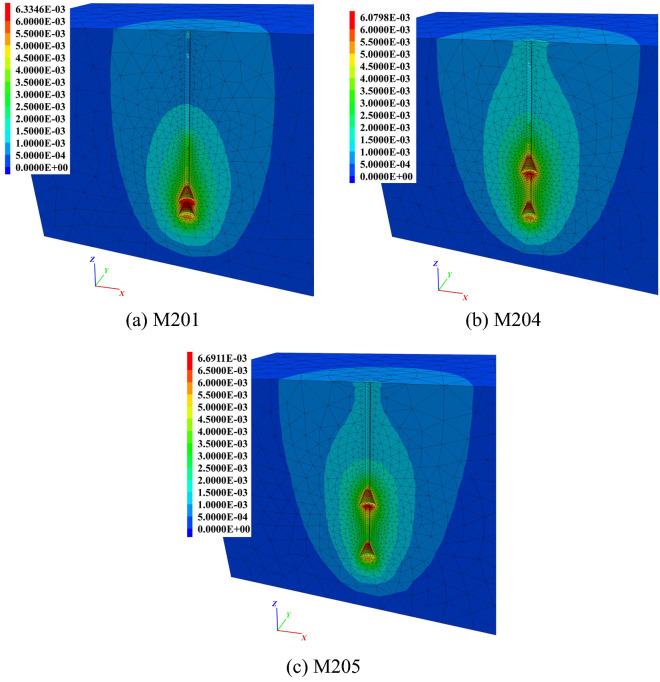
Soil vertical displacement contours of group M2 at 1.0*t*. (**a**) M201. (**b**) M204. (**c**) M205.

For easy comparison, 2-bell shaped underreams similar to M101 were adopted in group M2 of each anchor, M201–M205. The only difference among these 6 anchors is the spacing between bells. The single conical underream was 0.8 m in height, with a maximum diameter of 0.8 m. The length of the free section was 4 m, which equalled the anchorage length, and this setup was the same as group M1. The details can be found in Fig. 13. For space limitations, the soil vertical displacement contours of 3 anchors (M201, M204 and M205) in group M2 are shown in Fig. 14, and M101’s can be found in Fig. 11a.

In Figs. 11a and 14, it can be seen that in group M2, the soil vertical impact areas of M204 and M101 were relatively larger than those of the other 2 anchors. Because of the short spacing between neighbouring bells, the soil impact area around the middle shaft of anchor M201 was considerably larger than others. The potential fracture interfaces located at underreamed sections of M201 were in an approximately cylindrical shape. However, as the spacing of the bells increases, the potential fracture interface shrinks.

As the bell spacing continued to expand, the light yellow colour between the neighbouring bells became lighter, as shown by M204 in Fig. 14b. When the spacing of bells reached 2.0 m (M205), the light yellow colour at this area almost faded away. This implied that the grout–soil fracture interface of the middle shaft with anchor M205 had been formed at 1.0*t*. From the analysis above, it can be concluded that only when the bells and middle shaft were working at the same time could the greatest soil vertical displacement impact area around the anchor be observed.

**Figure 15:**
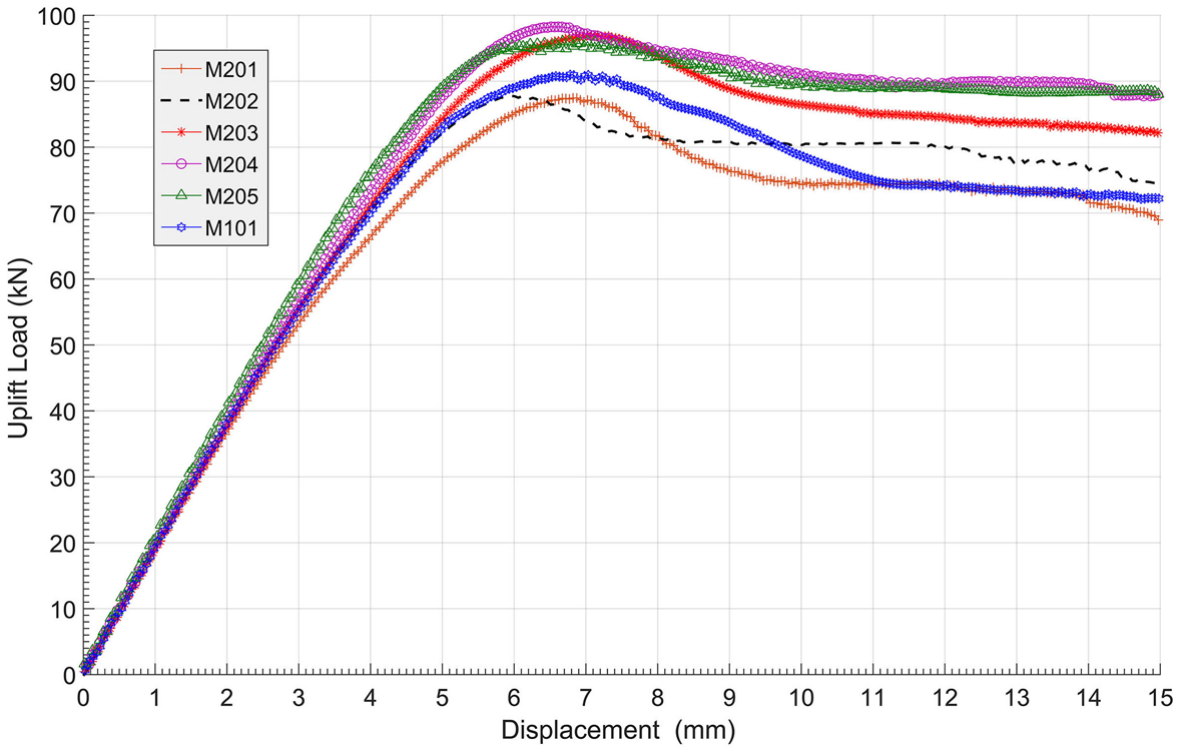
Load–displacement curves of group M2.

The load‒displacement curves of group M2 are shown in Fig. 15, and the related data are shown in Table 5. It can be observed directly in Fig. 15 that M203, M204 and M205 were the top 3 anchors in ultimate uplift bearing capacities, all above 96 kN. Meanwhile, M201 and M202 were the smallest two; due to the bells being set too close, the middle shaft resistance and ‘shoulder’ effect of the down bells were both not fully mobilized. From the analysis of these 6 anchors, proper expansion of the spacing between bells could efficiently promote anchor ultimate bearing capacity by 12.3% (M204 compared to M201) without incurring more concrete usage. However, if the spacing of bells became too sparse, such as M205, then the bearing capacity would drop. Therefore, there should be a critical value for reasonable spacing between anchor bells to achieve the optimal anti-uplift structure.

**Table 5:** Ultimate uplift bearing capacities and vertical displacement of group M2.

Anchor No	Ultimate uplift bearing capacity (kN)	Anchor vertical displacement (mm)	Promotion from M201 (%)
M201	87.5	6.8	0
M202	87.7	6.1	0.2
M101	89.0	7.4	1.7
M203	97.0	7.2	10.9
M204	98.3	6.5	12.3
M205	96.0	7.0	9.7

All load‒displacement curves in Fig. 15 behaved in similar patterns of increasing almost linearly first, slowing down in the development of resistance before reaching their summits, decreasing afterwards, and then staying stable. The reduction levels after reaching the summits of anchors M101, M201 and M202 were relatively greater than those of the other anchors. This indicates that expanding the bell spacing could not only enhance the ultimate uplift bearing capacity but also promote the residual resistance after reaching their peaks.

**Figure 16:**
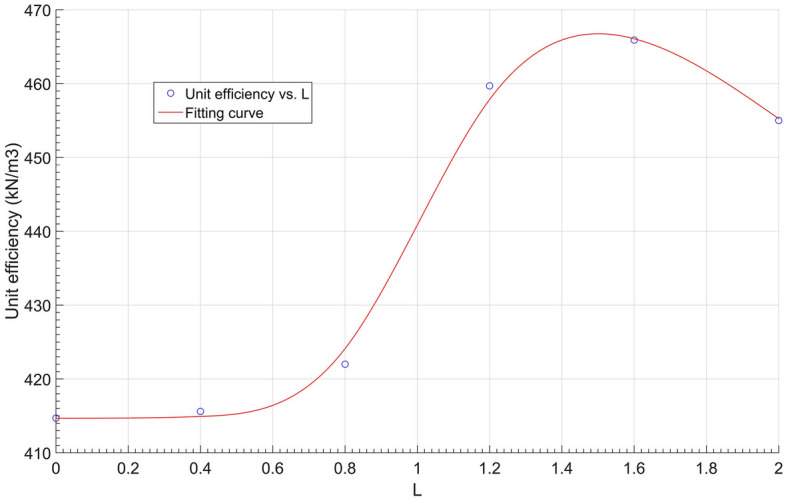
Anchorage efficiency vs. bell spacing curve of group M2.

Since all the anchors in group M2 shared the same body volume of 0.211 m^3^, the anchorage efficiency of each anchor is calculated in Table 6, and an anchorage efficiency vs. bell spacing curve produced from Table 6 is shown in Fig. 16. As seen in Fig. 16, when the bell spacing (*L*) was within the range of [1.3 m, 1.8 m] in group M2, the anchorage efficiency was above 460 kN/m^3^, providing the best performance for multibell anchors.

**Table 6:** Anchorage efficiency of group M2 anchors.

Dimensions (m)	Anchor no.					
	M201	M202	M101	M203	M204	M205
Bell spacing *L* (m)	0	0.4	0.8	1.2	1.6	2.0
Anchorage efficiency (kN/m^3^)	414.7	415.6	422.0	459.7	465.9	455.1

### The influence of bell underream diameter on ultimate uplift bearing capacity (Group M3)

**Figure 17:**
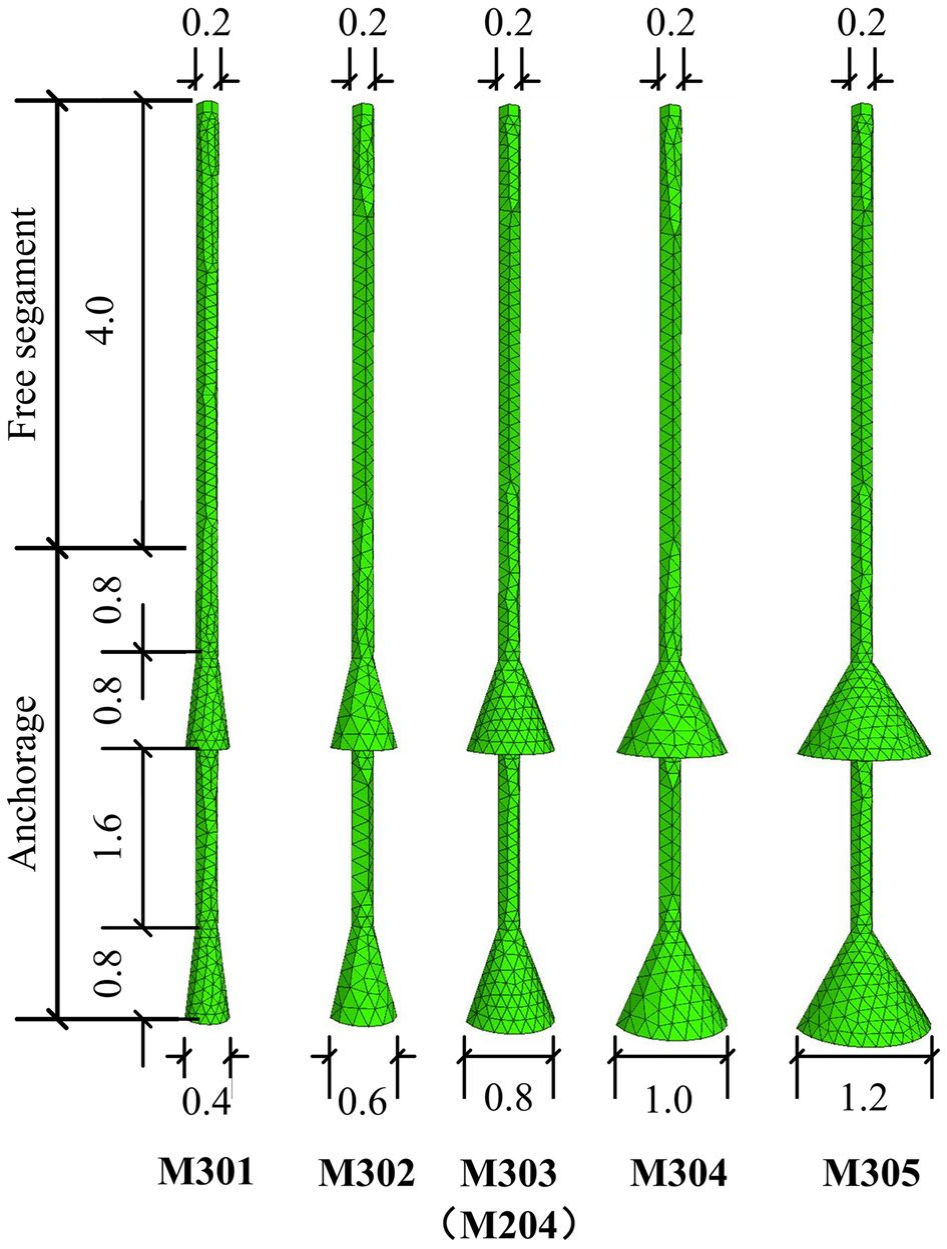
Diagram of group M3 anchors (Unit: m).

Five anchors were chosen with different underream diameters in group M3, and the details are shown in Fig. 17. The free section and anchorage height were both kept at 4 m, the same as group M1 and group M2. The surface area and concrete usage values were calculated and are shown in Table 7.

**Table 7:** Geometric parameters of group M3 anchors.

Anchor no.	M301	M302	M303 (M204)	M304	M305
Superficial area (m^2^)	2.59	3.18	3.86	4.64	5.53
Concrete volume (m^3^)	0.096	0.145	0.211	0.294	0.393

**Figure 18:**
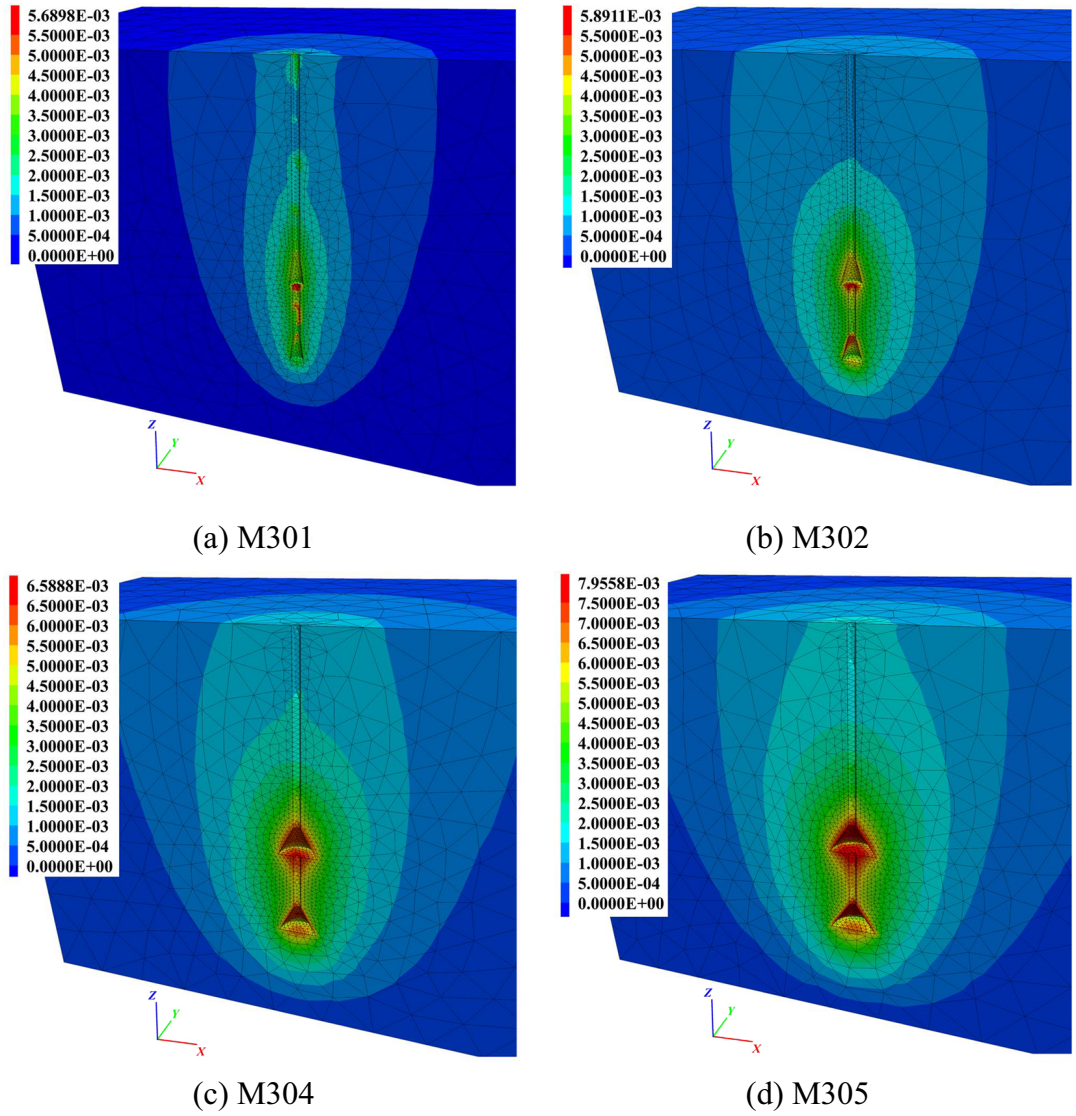
Soil vertical displacement contours of group M3 at 1.0*t*. (**a**) M301. (**b**) M302. (**c**) M304. (**d**) M305.

The soil vertical displacement contours of each anchor in group M3 are shown in Fig. 18 at 1.0*t*. It can be clearly observed in Fig. 18 that with an increase in the diameter of the underream, a larger impact area in the soil vertical displacement and a stronger ‘shoulder’ effect of each bell were observed. The soil impact area under the top bell was mainly governed by the bell’s dimensions, as seen clearly in Fig. 18c,d. The soil behaviour was the same as that of the soil under the anchor bottom, which flowed to fill the vacuum space created by uplifting the anchor. The wider the bell was, the more soil was disturbed under the bell.

Comparing Fig. 18a,b, different soil displacement patterns could be observed near the anchor bodies. In Fig. 18a, the same magnitude of soil displacements was activated close to the top shaft, conical surface and middle shaft of M301. This suggested that when the bell on the anchor body was not large enough, the anchor could not take advantage of the ‘shoulder’ effect and therefore did not perform as efficiently as a multibell structure. However, in Fig. 18b, the soil displacements surrounding the conical surfaces were greater than those around the shaft of M302, indicating that the potential yield interface had already been formed around the shaft.

On the other hand, different soil displacement contour patterns were observed in Fig. 18c,d. Because of the relatively larger bells, clearly defined potential fracture interfaces could not be found close to the middle shaft at 1.0*t*. In this case, with the same bell spacing, a more extensive impact area was created around the middle shaft during uplifting due to the larger diameter of the bells. This also expanded the potential yield interface, thus enhancing the ultimate uplift bearing capacity.

**Figure 19:**
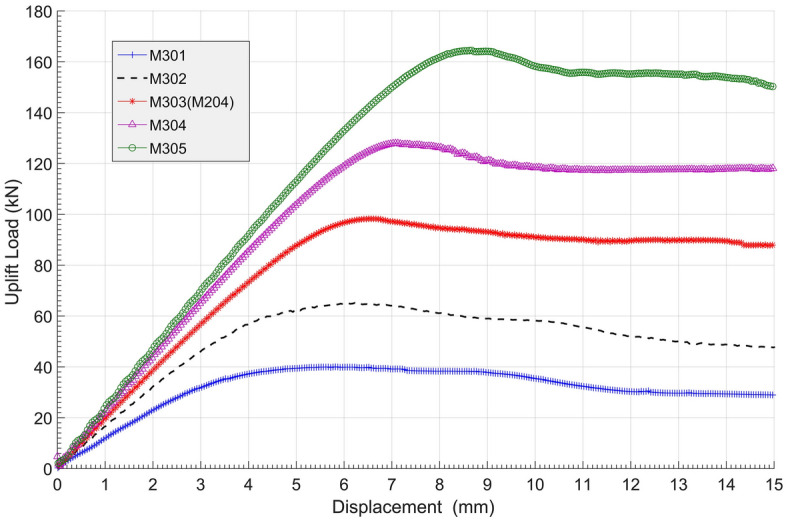
Load–displacement curves of group M3.

The load‒displacement curves and ultimate bearing capacity details of group M3 are shown in Fig. 19 and Table 8, respectively. The results show that a larger bell diameter led to a much higher anchor resistance. The lowest ultimate bearing capacity was M301 at 40 kN, but M305 had an ultimate bearing capacity of 164.4 kN with a 311% promotion compared to M301. Although all the load‒displacement curves decreased towards stable levels after reaching their own peaks, the extents of decline were different. Larger bell diameters resulted in a faster rate of reduction in loading capacities. Taking M301 and M305 as examples, the reduction after the peak was very obvious with M305, but M301 took nearly 6 mm to complete its postpeak decline. In another aspect, the increase in bell diameters could also extend the displacement before anchorage failure. From Table 8, the pull-out displacements of M301 and M305 were 5.8 mm and 8.7 mm, respectively, at 1.0*t*.

**Table 8:** Ultimate uplift bearing capacities and vertical displacements of group M3.

Anchor no.	Ultimate uplift bearing capacity (kN)	Anchor vertical displacement (mm)	Promotion from M301 (%)
M301	40.0	5.8	0
M302	65.0	6.1	62.5
M303 (M204)	98.3	6.5	145.8
M304	128.1	7.1	220.3
M305	164.4	8.7	311.0

### Discussion on the multibell anchor optimal structur*e*

**Figure 20:**
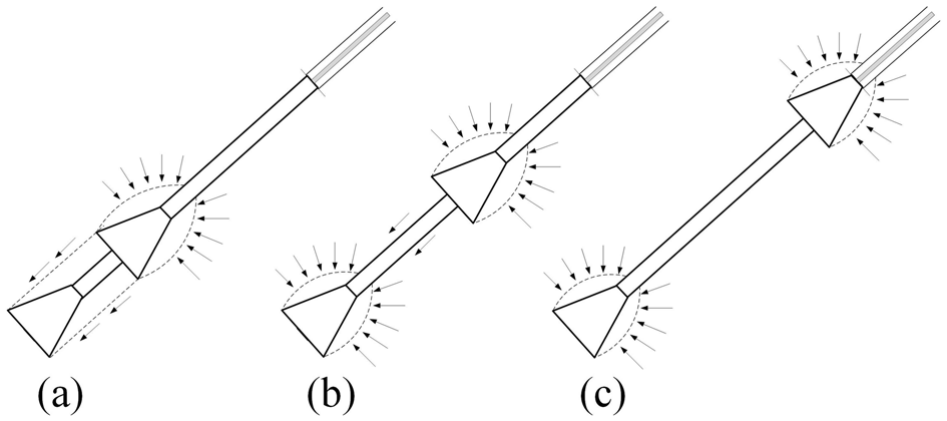
Anti-uplift mechanism of multibell anchor pulling out failure.

The analysis above suggested that the multibell anchor pull-out mechanism can be divided into three modes, as shown in Fig. 20, and it can also be seen that mode b (Fig. 20b) has the highest anchorage efficiency. To obtain the optimal structure of a multibell anchor, it is necessary to further investigate the relationship between them to obtain useful insights for geotechnical practice.

**Figure 21:**
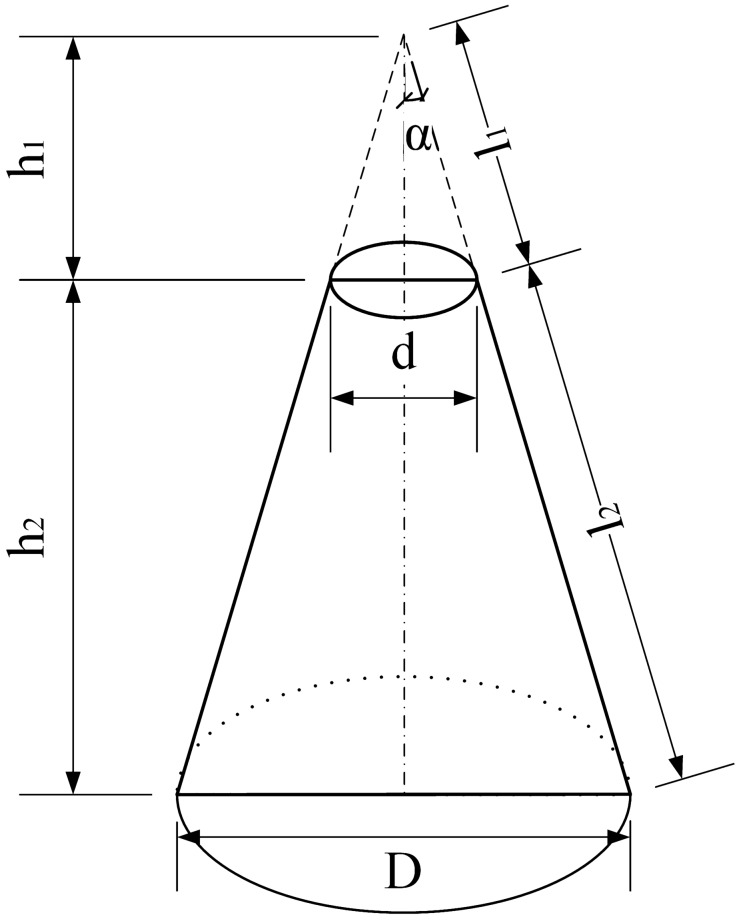
Calculation principle of bell superficial area.

For multibell anchors, the shear resistance around the top shaft already failed before the whole anchor reached the ultimate bearing capacity. The end bearing capacity from the ‘shoulder’ and shear resistance of the shaft were two main factors contributing to the ultimate uplift bearing capacity. In general, when the two parts were working together, the anchorage efficiency was the highest, as shown in Fig. 20. In other words, to achieve the highest anchorage efficiency, the ratio of ‘shoulder’ end bearing capacity to shaft shear resistance needs to be properly set. Taking M101 as an example, a coefficient $\lambda$ is introduced to represent the area ratio of the conical surface to the middle shaft. The calculation principle of the conical surface area is explained in Fig. 21 as ${S}_{1}=\pi /2\left[D\left({l}_{1}+{l}_{2}\right)-d{l}_{1}\right]$, and the shaft area is ${S}_{2}=\pi dL$, so $\lambda$ can be written as Eq. (1) as follows:

1 $$\lambda =\frac{{S}_{1}}{{S}_{2}}=\frac{{D}^{2}-{d}^{2}}{4dL\ \mathrm{sin}\alpha }.$$

**Figure 22:**
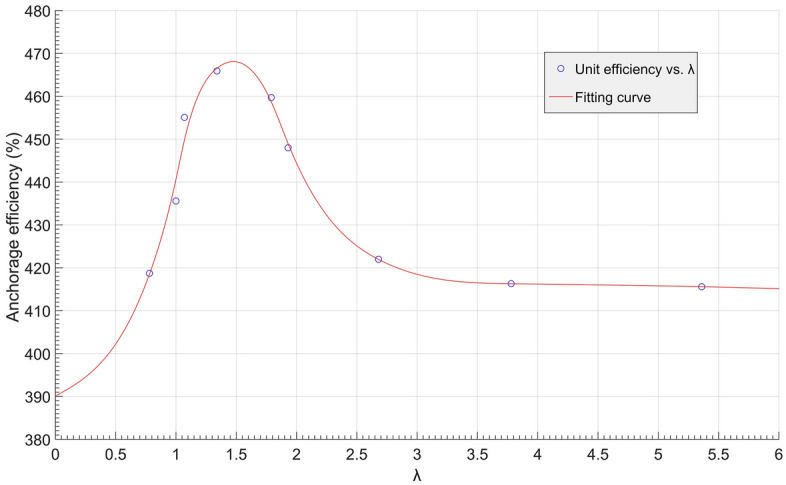
Anchorage efficiency vs. $\uplambda$ curve.

To find the optimal multibell anchor structure, some simulated anchors with associated parameters are listed in Table 9, and a curve of the anchorage efficiency vs. the $\lambda$ value is plotted in Fig. 22. In the table, the anchors presented were chosen from groups M2 and M3 plus another from group M1. All anchors shared the same anchorage length (4.0 m), and their ratios of anchorage efficiency to concrete usage were calculated and analysed.

**Table 9:** Anchorage efficiency anchors.

Anchor No	$\lambda =\frac{{D}^{2}-{d}^{2}}{4dL\mathrm{sin}\alpha }$	Concrete volume (m^3^)	Anchorage efficiency (kN/m^3^)
M201	–	0.211	414.7
M202	5.36	0.211	415.6
M101	2.68	0.211	422.0
M203	1.79	0.211	459.7
M204	1.34	0.211	465.9
M205	1.07	0.211	455.1
M301	3.78	0.096	416.3
M302	1.93	0.145	448.0
M304	1.00	0.294	435.6
M305	0.78	0.393	418.7

A notable peak can be noted in Fig. 22, and its value is approximate at 470 kN/m^3^, while $\lambda$ equals 1.5. It can be concluded that when $\lambda \in [1, 1.8]$, the anchorage efficiency can be maintained above 450 kN/m^3^, while the multibell anchor can perform the best by saving concrete usage.

## Conclusions

Based on synthetic transparent clay and the PIV technique, pull-out model tests on multibell soil anchors were undertaken. Numerical simulations with three groups of anchors M1 (different shapes of bells), M2 (different bell spacings) and M3 (different bell diameters) were studied. The soil vertical displacement contours and anchor load‒displacement curves were analysed to investigate the anchorage mechanism and optimal structure of the soil anchor. The following was determined:

1. While keeping the other dimensions unchanged, varying the concave–convex curvature of the bell surface could significantly improve the ultimate uplift bearing capacity of the anchor. A convex conical bell surface could enhance the bearing capacity more obviously than a concave conical surface, but it would increase concrete usage, therefore reducing the anchorage efficiency.
2. If the bell shape and other dimensions were the same, there was a critical value for the spacing of bells, allowing the multibell anchor to reach the highest ultimate bearing capacity.
3. There are three modes of the multibell anchor pull-out mechanism, and the ‘shoulder’s’ end bearing and shaft resistance should be designed systematically to achieve the best anchorage performance.
4. An associated coefficient $\lambda$ was introduced to evaluate the anchorage efficiency of multibell anchors. When $\lambda$ is approximately 1.5, the anchorage efficiency achieves the highest value.

### Limitations

Due to transparency problems, the confined space of experiment perspex box may cause boundary effect and can be improved in future research. The press plate with a hole may cause diffusion effect of the soil and affect the test results.

## Data Availability

All data generated or analysed during this study is available from the corresponding author on reasonable request.
