# Author Correction: Structural parameter variation working on the performance of anti-uplift multibell underreamed anchors

**DOI:** 10.1038/s41598-022-23192-y

**Published:** 2022-11-04

**Authors:** Chen Chen, Yuanyou Xia, Manqing Lin, Qing Ni

**Affiliations:** 1 School of Civil Engineering and Architecture, Wuhan Institute of Technology, Wuhan, 430073 China; 2 School of Civil Engineering and Architecture, Wuhan University of Technology, Wuhan, 430070 China; 3 School of Resources and Safety Engineering, Wuhan Institute of Technology, Wuhan, 430073 China; 4 School of Engineering, University of Warwick, Coventry, CV4 7ES UK

Correction to: *Scientific Reports* 10.1038/s41598-022-19353-8, published online 02 September 2022

The original version of this Article contained an error in the Acknowledgements section.

“This study is supported by the National Natural Science Foundation of China (NSFC) with Nos: 52174085 and 51374163.”

now reads:

"This study is supported by the National Natural Science Foundation of China (NSFC) with Nos: 52278369, 52174085 and 51374163."

The original Article has been corrected.

